# N^6^‐methyladenosine‐mediated upregulation of LNCAROD confers radioresistance in esophageal squamous cell carcinoma through stabilizing PARP1

**DOI:** 10.1002/ctm2.70039

**Published:** 2024-10-05

**Authors:** Xiaobo Shi, Xiaozhi Zhang, Xinran Huang, Ruijuan Zhang, Shupei Pan, Shan Huang, Yuchen Wang, Yue Ke, Wei Guo, Xiaoxiao Liu, Yu Hao, You Li, Xu Zhao, Yuchen Sun, Jing Li, Hongbing Ma, Xixi Zhao

**Affiliations:** ^1^ Department of Radiation Oncology The Second Affiliated Hospital of Xi'an Jiaotong University Xi'an China; ^2^ Department of Radiation Oncology The First Affiliated Hospital of Xi'an Jiaotong University Xi'an China; ^3^ Department of Peripheral Vascular The First Affiliated Hospital of Xi'an Jiaotong University Xi'an China

**Keywords:** esophageal squamous cell carcinoma, LNCAROD, N6‐methyladenosine, PARP1, radioresistance

## Abstract

**Background:**

Radiotherapy is a primary therapeutic modality for esophageal squamous cell carcinoma (ESCC), but its effectiveness is still restricted due to the resistance of cancer cells to radiation. Long non‐coding RNAs (lncRNAs) and N^6^‐methyladenosine (m6A) have been shown to play significant roles in tumour radioresistance. However, the precise manifestation and role of m6A‐modified lncRNAs in ESCC radioresistance remain unclear.

**Methods:**

Bioinformatics analysis was conducted to identify m6A‐modified lncRNAs implicated in the radioresistance of ESCC. A series of functional experiments were performed to investigate the function of LNCAROD in ESCC. Methylated RNA immunoprecipitation, chromatin isolation by RNA purification‐mass spectrometry, RNA immunoprecipitation, and co‐immunoprecipitation experiments were performed to explore the mechanism of m6A‐mediated upregulation of LNCAROD expression and the downstream mechanism enhancing the radioresistance of ESCC. The efficacy of LNCAROD in vivo was assessed using murine xenograft models.

**Results:**

Herein, we identified LNCAROD as a novel METTL3‐mediated lncRNA that enhanced radioresistance in ESCC cells and was post‐transcriptionally stabilised by YTHDC1. Moreover, we confirmed that LNCAROD prevented ubiquitin‐proteasome degradation of PARP1 protein by facilitating PARP1‐NPM1 interaction, thereby contributing to homologous recombination‐mediated DNA double‐strand breaks repair and enhancing the radiation resistance of ESCC cells. Silencing LNCAROD in a nude mouse model of ESCC in vivo resulted in slower tumour growth and increased radiosensitivity.

**Conclusion:**

Our findings enhance the understanding of m6A‐modified lncRNA‐driven machinery in ESCC radioresistance and underscore the significance of LNCAROD in this context, thereby contributing to the development of a potential therapeutic target for ESCC patients.

## Graphical abstract

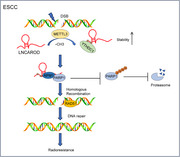

## 1 BACKGROUND

Esophageal cancer, a common malignant tumour of the digestive tract, ranks sixth in the world for death and seventh in terms of incidence.^1^ The major pathological subtypes of esophageal cancer include esophageal adenocarcinoma and esophageal squamous cell carcinoma (ESCC), with ESCC accounting for approximately 90% of the histological classifications in China.^2^, ^3^ Due to the insidious onset and rapid growth of ESCC, most patients are already in the advanced stage upon seeking medical treatment, with radiotherapy being their primary therapeutic modality.^4^ Despite remarkable advances in radiotherapy techniques, a considerable proportion of patients still encounter local recurrence due to radioresistance, and the 5‐year survival rate for patients is still less than 30%.^5^ Hence, it is imperative to clarify the molecular mechanisms underlying radioresistance, providing novel insights for optimising radiosensitisation therapy in ESCC.

N^6^‐methyladenosine (m6A) is a prevalent modification observed in eukaryotic cells during the posttranscriptional stage, exerting significant influence on RNA stability, alternative splicing, subcellular localisation and translation efficiency.^6^ The m6A modification of RNAs is primarily deposited by the m6A methyltransferase complex, which is composed of METTL3, METTL14 and WTAP, and reversed by m6A demethylases, such as ALKBH5 and FTO, maintaining its dynamic balance.^7^, ^8^, ^9^, ^10^, ^11^ The m6A modification is detected by m6A reader proteins, including YTHDC1/2, IGF2BP1/2/3 and others, to execute a corresponding regulatory function.^12^, ^13^ Emerging evidence indicates that m6A modification can influence the expression and function of genes involved in DNA repair pathways, thereby affecting the efficiency of repair following ionising radiation (IR)‐induced damage.^14^ Recently, Zhang et al. reported that m6A‐modified RNAs form RNA‐DNA hybrids at double‐strand DNA breaks (DSBs) sites and then promotes the recruitment of RAD51 and BRCA1 to the DSB sites, facilitating homologous recombination (HR) repair.^15^ So far, most of the research on tumour radioresistance has primarily concentrated on the impact of m6A modification on mRNAs.^16^ The precise mechanism by which m6A modification regulates long noncoding RNAs (lncRNAs) in ESCC resistance to radiation still remains elusive.

Here, we have found that METTL3 facilitates the m6A modification on lncRNA activating regulator of DKK1 (LNCAROD), which is subsequently recognised by YTHDC1, thereby regulating the stability of LNCAROD. LNCAROD is upregulated in ESCC and increases the resistance of ESCC cells to radiation. Mechanistically, LNCAROD functions as a scaffold that promotes the interaction between PARP1 and NPM1 proteins, thereby impeding the ubiquitin‐proteasome degradation of PARP1 protein. Moreover, we found LNCAROD promotes HR‐mediated DNA repair by maintaining PARP1 protein stability, thereby alleviating the lethal effects on ESCC cells under radiation exposure. Our findings enhance the understanding of m6A‐modified lncRNA‐driven machinery in ESCC radioresistance and underscore the significance of LNCAROD in this context, thereby facilitating the identification of a potential therapeutic target for ESCC patients.

## 2 METHODS

### 2.1 LncRNA expression profile

The transcription profile and relevant clinical data of ESCC patients were obtained from The Cancer Genome Atlas (TCGA) database (Table S1). The dataset of gene expression microarray (GSE53625), which includes 179 patients with ESCC, was obtained from the Gene Expression Omnibus database (GEO) (Table S2). Differentially expressed lncRNAs were identified as previously described.^17^

### 2.2 Cell culture and reagents

The human ESCC cell lines, including KYSE‐150, KYSE‐30, KYSE‐410, and TE‐1, were acquired from Procell Life Science & Technology (Procell, WuHan, China). The Het‐1A cell line, derived from human normal esophageal epithelial cells, was available from the American type culture collection (ATCC, Maryland, USA). Radioresistant cells (KYSE‐150R) were generated from parental KYSE‐150 cells as previously described.^18^, ^19^ The KYSE‐150 and KYSE‐150R cell lines used in our study were authenticated by short tandem repeat (STR) analysis prior to experimentation. The STR profiling confirmed the integrity and identity of the cell lines, ensuring that no cross‐contamination or genetic drift occurred during the development of the resistant cells. Actinomycin D and proteasome inhibitor MG132 were acquired from MedChemExpress (New Jersey, USA), while cycloheximide (CHX), a protein synthesis inhibitor, was obtained from Selleck Chemicals (Houston, TX, USA). In this study, a Versa HD medical linear accelerator, manufactured by Elekta in Sweden, was utilised as the X‐ray source. The experiment employed 6 MV X‐rays, with the irradiation dose adjusted according to the specific experimental requirements. The cell samples were positioned on the accelerator's treatment couch, maintaining a source‐to‐skin distance (SSD) of 100 cm.

### 2.3 Plasmids, lentivirus and transfection

Antisense oligonucleotides (ASOs) targeting LNCAROD (ASO‐LNCAROD#1 and ASO‐LNCAROD#2) and control ASO (ASO‐NC) were synthesised from RiboBio (Guangzhou, China). Small interfering RNAs (siRNAs) against YTHDC1 (si‐YTHDC1), NPM1(si‐NPM1) and negative control siRNA (si‐NC) were purchased from Sangon (Shanghai, China). Short hairpin RNA (shRNA) against METTL3 (sh‐METTL3), shRNA negative control (sh‐NC) lentivirus, PARP1 overexpression plasmid (PARP1), METTL3 overexpression plasmid (METTL3) and empty vector (vector) were obtained from GeneChem (Shanghai, China). The CRISPR‐Cas9 lentiviral vectors for knockout LNCAROD (KO‐LNCAROD#1 and LNCAROD#2) were constructed by TsingKe Biotech (Beijing, China). The ESCC cells were subjected to transfection with ASOs, siRNA, or overexpression plasmids using the Lipofectamine 3000 transfection kit (Invitrogen) following the guidelines provided by the manufacturer. The lentiviral transduction procedure was executed following the guidelines provided by the manufacturer, followed by a 2‐week cell screening using puromycin. After confirming the accuracy of the sequence, the stable transduction cells were diluted, selected and cultured to obtain specific stable cells. The target sequences used in this study are shown in Table S3.

### 2.4 RNA extraction and quantitative reverse transcription polymerase chain reaction (RT‐qPCR)

The cultured cells were subjected to TRIzol (TaKaRa, Dalian, China) for total RNA isolation. Subsequently, the Prime Script™ RT reagent kit (TaKaRa) was used for cDNA synthesis, followed by qPCR analysis utilising TB Green® Premix Ex Taq™ II (TaKaRa). Table S4 presents the primer sequences.

### 2.5 Western blot

We used a lysis buffer to extract total protein from ESCC cells and employed the Cell Nuclear and Cytoplasmic Protein Extraction Kit (Beyotime, Shanghai, China) to separate extraction of cytoplasmic and nuclear proteins. Afterwards, we quantified the protein using the BCA Protein Assay Kit (Beyotime). Equal amounts of protein obtained from the experimental samples were loaded onto SDS‐PAGE gels, electrophoresed, and transferred to a PVDF membrane (Millipore, Billerica, MA, USA). The membranes were blocked with 5% non‐fat milk before overnight incubation with specific primary antibodies at 4°C. After washing, the corresponding secondary antibodies were applied. The chemiluminescence kits (Millipore, USA) were used for visualising the protein bands. β‐Tubulin was utilised as an internal reference to normalise total protein and cytoplasmic proteins, whereas Histone H3 (H3) was employed as an internal reference for nuclear proteins. Antibodies were shown in Table S5.

### 2.6 Total RNA m6A quantification

Total RNA was extracted from cellular samples and subjected to quantitative m6A analysis using the Methylation Quantification Kit (Colorimetric) from Epigentek, New York, USA. Briefly, the assay utilised a specific m6A antibody for RNA capture, followed by colorimetric readout. The procedure involved immobilising RNA, blocking non‐specific binding, incubating with the antibody, and detecting enzymatically. Absorbance readings at 450 nm were directly correlated with m6A levels quantified against standard curves.

### 2.7 Methylated RNA immunoprecipitation (MeRIP) assay

The EpiQuikTM CUT&RUN m6A RNA Enrichment (MeRIP) Kit (EpiGentek, USA) was employed for the identification of m6A enrichment on the LNCAROD transcript. 10 µg of total RNA was collected, cleaved, incubated with corresponding antibodies, purified and eluted to obtain coprecipitated RNA samples with m6A modification sites for RT‐qPCR detection. SRAMP predicted the m6A modification site in the LNCAROD transcript, which was the basis for the precise design of the primers for MeRIP‐qPCR(https://www.cuilab.cn/sramp).^20^ Table S4 displays the primer sequences.

### 2.8 Fluorescence in situ hybridisation (FISH)

The cells were initially dispersed in a 24‐well plate at a concentration of 40% and incubated overnight. After a PBS wash and fixation with 4% paraformaldehyde, the cells were treated overnight at 37°C in the dark with specific probes (LNCAROD, 18S rRNA, or U6 RNA). Then, the cells underwent sequential treatment with different washing buffers at room temperature. Finally, fluorescence microscopy was used to capture images after DAPI staining lasting for 10 min.

### 2.9 Isolation of cytoplasmic and nuclear RNA

Following the manufacturer's instructions, the Cytoplasmic & Nuclear RNA Purification Kit (Norgen Biotek) was used to separate cytoplasmic and nuclear RNA from KYSE‐150 and TE‐1 cells. The nuclear indication was HOTAIR, while the cytoplasmic reference was GAPDH. Table S4 displays the primer sequence.

### 2.10 Colony formation, cell cycle, cell apoptosis and immunofluorescence staining

The detailed protocols were similarly described as previously.^18^

### 2.11 RNA stability analysis

The ESCC cells that underwent transfection were meticulously prepped and placed in a 6‐well plate, with each well containing about 3 × 10^5^ cells. Subsequently, the cells were cultured overnight and were subjected to treatment with Actinomycin D (ActD) for the designated time. The RT‐qPCR technique was used to extract and evaluate the expression levels of LNCAROD in each group.

### 2.12 RNA immunoprecipitation (RIP)

The manufacturer's protocol was followed to perform RIP experiments using the RIP RNA‐Binding Protein Immunoprecipitation Kit (BersinBio, China). Briefly, cultivate cells, lyse them using BersinBio RIP Kit's lysis buffer with inhibitors. Quantify protein levels and immunoprecipitate with antibodies targeting the RNA‐binding protein of interest using magnetic beads. Thoroughly wash the beads, elute RNA, and extract it for RT‐qPCR. The antibodies are detailed in Table S5.

### 2.13 Chromatin isolation by RNA purification (ChIRP)‐mass spectrometry (MS)

To screen proteins that bind to LNCAROD, we designed 10 probes covering the LNCAROD sequence for ChIRP experiments. U1 snRNA probe was used as positive control. ChIRP‐MS analysis was carried out by aksomics (Shanghai, China). In brief, 2 × 10^7^ cells were subjected to reversible formaldehyde crosslinking in situ and hybridised with biotin‐labelled probes. Subsequently, the cells underwent vigorous denaturation conditions to remove non‐specifically bound proteins. Finally, mass spectrometry was employed for the identification and quantitative analysis of proteins that specifically interacted with LNCAROD or the control.

### 2.14 Co‐immunoprecipitation (co‐IP)

We cultured and collected 1 × 10^7^ ESCC cells for IP experiments. Cells were then lysed using IP lysis solution (Beyotime, Shanghai, China) containing PMSF, a protease inhibitor, and a phosphatase inhibitor. After centrifugation, we collected the lysates from the cells and allowed them to incubate overnight at a temperature of 4°C with the corresponding antibodies. Subsequently, we resuspended Pierce™ Protein A/G Magnetic Beads (Thermo Scientific™) in the cell lysates and subjected them to a 3 h incubation at 4°C on a rotary shaker. Following, we performed protein elution using an elution buffer after washing the beads with a wash buffer. Finally, western blot assay was used to detected these proteins. The antibodies are detailed in Table S5.

### 2.15 Protein stability analysis

The transfected ESCC cells were placed in a 6‐well plate, with an approximate cell count of 3 × 10^5^ cells per well. Subsequently, the cells were cultured overnight and were subjected to treatment with CHX for the specified durations. Relevant proteins from each group were isolated and analysed using western blot technique.

### 2.16 Neutral comet assay

Cells were exposed to radiation (8 Gy) and harvested at certain time intervals after irradiation. According to the manufacturer's instructions, neutral comet experiments were conducted using the comet Assay Kit (KeyGEN, Nanjing, China). First, we coated a glass slide with 120 µL of melted 1% normal melting agarose and then placed it at 4°C until it solidified. Second, the single‐cell suspension was mixed with ice‐cold PBS to a final volume of 1×10^6^ cells/mL, and then, 10 µL of the suspension was dipped in 100 µL low melting agarose. Third, we carefully dispensed 110 µL of the single‐cell suspension onto the pre‐coated slide and swiftly distributed it uniformly. After the suspension was coagulated, we used a neutral lysis buffer to lyse the gel overnight in the dark. Then, the slides were electrophoresed at 4°C for 30 min at 25 V under neutral conditions. Finally, PI (10 µg/mL) was applied to the gels and left for 20 min. All of the gels were observed using fluorescence microscopy, and comet images from each slide were analysed using CASP (CASPlab, Wroclaw, Poland) to determine the tail moment.

### 2.17 Xenograft tumour mouse model and treatment

Female BALB/C nude mice, aged 4 weeks, were kept in a specified pathogen‐free (SPF) animal facility. The facility maintained a constant temperature of 22−25°C, with a 12‐h cycle of alternating day and night. Mice were randomly assigned, and 5×10^6^ ESCC cells were delivered subcutaneously to their right flanks. The digital calliper was used to measure the volume of the tumour at intervals of 3 days, commencing from day 7 after the injection of the tumour, and the calculation was performed using a specific formula: the maximum diameter (*L*) × minimum tumour diameter (*W*)^2^/2. Once the tumour volume reached a range of 50−100 mm^3^, the mice were subjected to either irradiation or no irradiation, using X‐rays specifically directed towards the tumour area (administered as 4 Gy daily for a duration of 3 days). On day 28 after injection, mice in the control group were euthanised directly, while mice in the treatment group were sacrificed after receiving 4 Gy of local irradiation. The tumours were surgically removed and then preserved in paraffin for the subsequent IHC analyses.

### 2.18 Immunohistochemistry (IHC)

The sections underwent deparaffinisation, permeabilisation, antigen retrieval and blocking. Subsequently, the samples were exposed to PARP1, γH2AX, and Ki67 antibodies and incubated overnight at a temperature of 4°C. Following this, the samples were rinsed with PBS three times. Afterwards, the sections were treated with a secondary antibody that was linked to HRP for a duration of 1 h at room temperature. They were then washed three times with PBS and underwent staining with DAB and hematoxylin. Ultimately, the sections underwent dehydration using different concentrations of ethanol and were thereafter mounted with coverslips. The SlideViewer system from 3DHISTECH was utilised to acquire images of the sections. Immunoreactive scores were calculated as described previously.^21^ The antibodies are found in Table S5.

### 2.19 Statistical analysis

The statistical analysis and data visualisation in the present work were conducted using the software GraphPad Prism 8.0 and the programming language R 4.2.1. A two‐tailed unpaired *t*‐test or *χ* ^2^ test was used in statistical analysis to assess differences between two groups. a one‐way ANOVA was employed for analyses involving more than two groups. The experimental results were reported as the mean value plus or minus the standard deviation (SD). A *p*‐value of less than  .05 showed statistical significance.

## 3 RESULTS

### 3.1 METTL3‐mediated m6A modification enhances the stability of LNCAROD in a YTHDC1‐dependent manner

**FIGURE 1:**
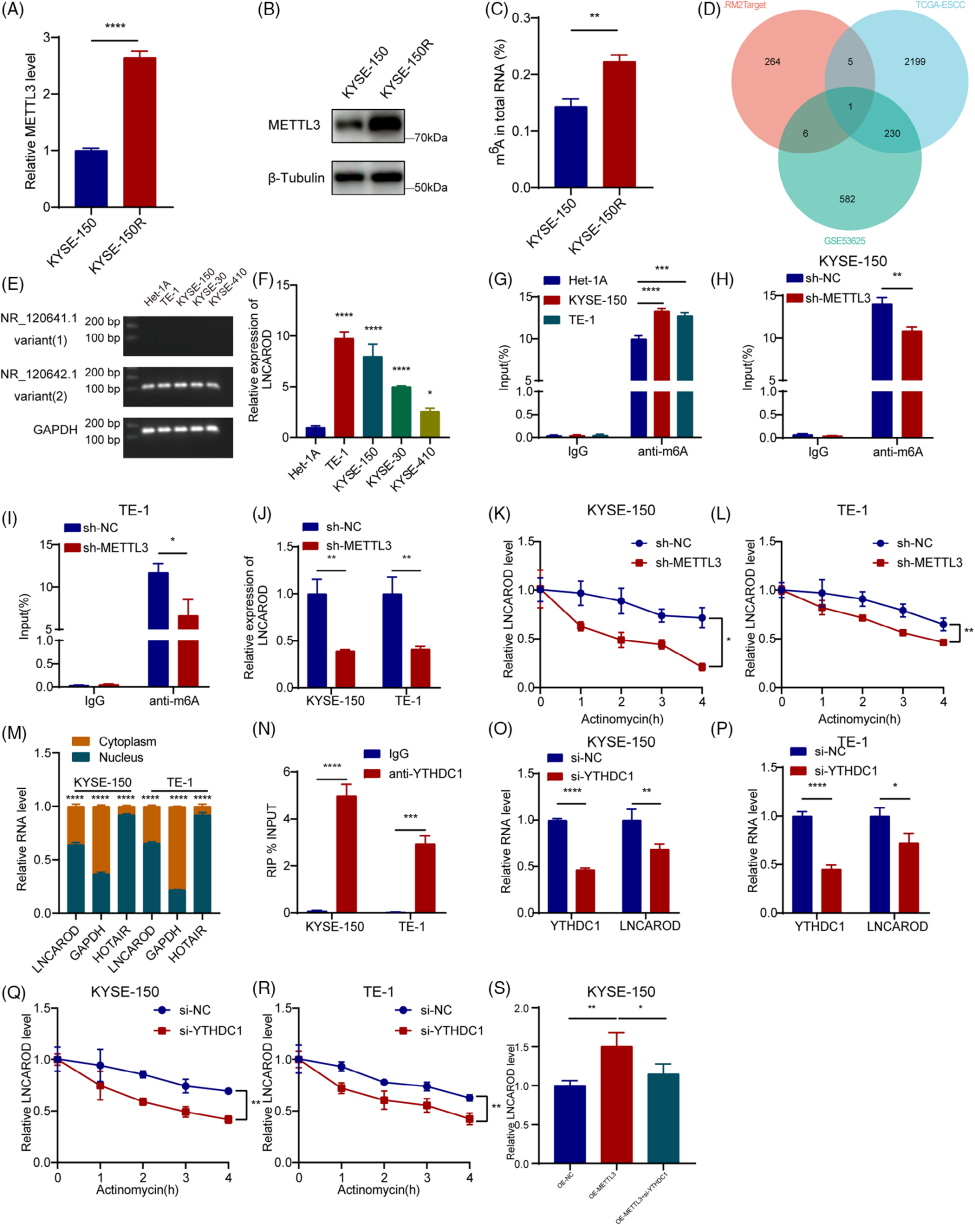
METTL3‐mediated m6A modification enhances the stability of LNCAROD in a YTHDC1‐dependent manner. (A, B) RT‐qPCR analysis and Western blot analysis for the expression of METTL3 mRNA and protein in parental and radioresistant ESCC cell lines. (C) Total m6A levels in radioresistant and parental ESCC cell lines. (D) The Venn diagram shows that LNCAROD is the only lncRNA modified by METTL3‐mediated m6A and overlapping in both GSE53625 and TCGA independent ESCC cohorts. (E) RT‐PCR assay is employed to assess the expression of LNCAROD variants in ESCC cell lines and normal cells. (F) Expression levels of LNCAROD in ESCC cell lines and normal cells is examined by RT‐qPCR assay. (G) MeRIP assay results show the enrichment of m6A‐modified LNCAROD in the indicated cells. (H, I) MeRIP‐qPCR analysis of LNCAROD m6A modification in the indicated cells. (J) RT‐qPCR analysis of LNCAROD expression in the indicated cells. (K, L) The RNA stability of LNCAROD is measured by RT‐qPCR in the indicated cells. Actinomycin D (5 mg/mL) is added at time 0. (M) Cell nuclear/cytoplasmic fractionation and RT‐qPCR show the cellular distribution of LNCAROD in ESCC cells. GAPDH is used as a cytoplasmic marker and HOTAIR is used as a nuclear marker. (N) RIP‐qPCR assays show the relative enrichment of LNCAROD detected by YTHDC1 antibody. (O, P) RT–qPCR assays show the relative expression of YTHDC1 and LNCAROD in the indicated cells. (Q, R) The RNA stability of LNCAROD is measured by RT‐qPCR in the indicated cells. (S) Knockdown of YTHDC1 significantly abolished the upregulation of LNCAROD induced by ectopic METTL3 expression in KYSE‐150 cells. Data are shown as the mean ± SD from three independent experiments (*p < .05, **p < .01, ***p < .001, ****p < .0001).

To evaluate the link between radioresistance and RNA m6A modification in ESCC, we examined the mRNA and protein expression levels of several dominant m6A methyltransferases (METTL3, METTL14 and WTAP) and demethylases (FTO and ALKBH5) in previously established parental and radioresistant ESCC cell lines. Both RT‐qPCR and Western blot analyses revealed a significant upregulation of METTL3 mRNA transcript level and protein abundance in radioresistant cells compared to parental cells (Figure 1A and B), while no alterations were observed in the expression of METTL14, WTAP, FTO, or ALKBH5 (Figure S1A). Furthermore, the overall level of m6A was elevated in radioresistant cells compared to parental cells, consistent with the upregulation of METTL3 expression in radioresistant cells (Figure 1C). Studies have shown that METTL3 is upregulated and has a carcinogenic function in ESCC.^22^, ^23^ This was also validated by the TCGA‐ESCC and GSE53625 datasets (Figure S1B and C). To further identify METTL3‐regulated lncRNAs, a Venn diagram was employed to intersect the molecules confirmed to be METTL3‐mediated m6A‐modified in the RM2Target database with differentially expressed lncRNAs in the TCGA‐ESCC and GSE53625 datasets (Table S6). The results showed that LNCAROD was the only lncRNA significantly elevated and METTL3‐mediated in ESCC tissue (Figure 1D). Further experiments revealed that only variant 2 of LNCAROD is expressed at a high level in a variety of ESCC cells (Figure 1E and F). We identified many m6A sites on LNCAROD using SRAMP (Figure S1D). Furthermore, MeRIP‐qPCR results indicated that the m6A level of LNCAROD in ESCC cells was significantly higher than in normal esophageal cells (Figure 1G). Subsequently, METTL3 expression in ESCC cell lines was detected using Western blot, and KYSE150 and TE‐1 cells with relatively high METTL3 expression were selected for METTL3 silencing (Figure S1E and F). Further experiments showed that knocking down METTL3 in ESCC cells significantly reduced the m6A level of LNCAROD (Figure 1H and I). Based on the above results, we confirmed that in ESCC cells, LNCAROD was regulated by METTL3‐mediated m6A modification. Moreover, the depletion of METTL3 resulted in a notable decrease in the expression level of LNCAROD in ESCC cells (Figure 1J). Thus, we propose that m6A may affect the stability of LNCAROD. To verify this hypothesis, we evaluated the effect of METTL3 on LNCAROD stability by actinomycin D RNA stability experiments and found that the half‐life of LNCAROD in METTL3 knockdown group was significantly shorter than that in NC group (Figure 1K and L).

Nuclear and cytoplasmic fractionation experiments and FISH detection revealed that LNCAROD was mainly localised in the nucleus (Figures 1M and S1G). The m6A modification of RNA is mainly recognised by m6A ‘reader’, thereby regulating RNA stability. Current studies suggest that YTHDC1 is located in the cell nucleus and may be involved in protecting m6A‐modified LNCAROD.^24^ Through RIP experiments, we validated the direct binding between YTHDC1 and LNCAROD (Figure 1N), and silencing YTHDC1 in ESCC cells significantly reduced LNCAROD level (Figure 1O and P). In the presence of actinomycin D, knockdown of YTHDC1 notably impaired the stability of LNCAROD (Figure 1Q and R). Furthermore, we confirmed that the deletion of YTHDC1 abolished the upregulation of LNCAROD induced by ectopic METTL3 expression in KYSE‐150 cells (Figure 1S). In summary, our findings indicated that METTL3‐mediated m6A modification of LNCAROD enhanced its stability in a YTHDC1‐dependent manner, which caused the upregulation of LNCAROD expression levels in ESCC cells.

### 3.2 LNCAROD enhances radioresistance of ESCC cells in vitro

**FIGURE 2:**
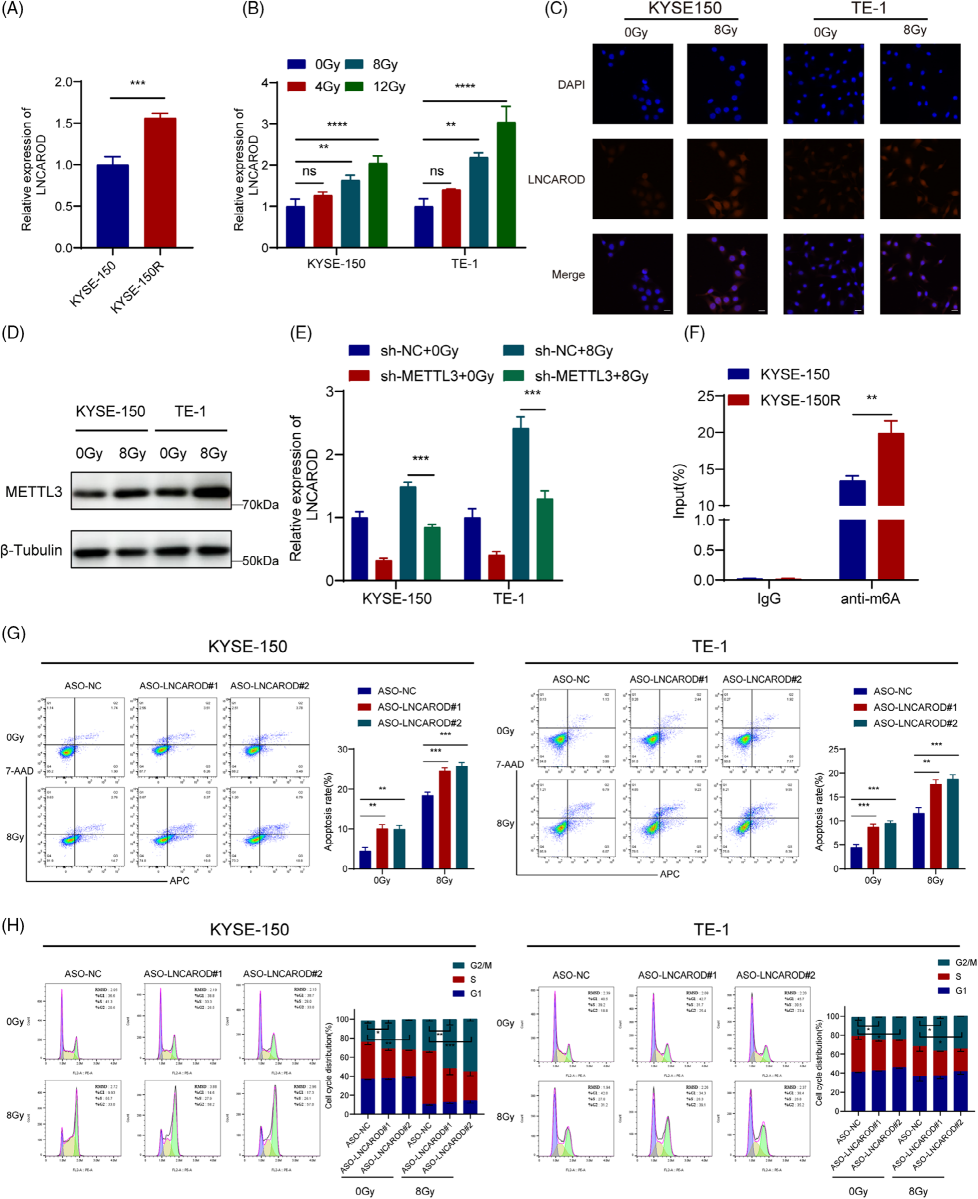
LNCAROD is regulated by METTL3 to affect the radioresistance of ESCC cells. (A) RT‐qPCR analysis of LNCAROD expression in radioresistant and parental ESCC cell lines. (B) RT‐qPCR analysis of LNCAROD expression in the indicated cells after treatment with a gradient of radiation doses. (C) FISH results for LNCAROD expression in the indicated cells following treatment with 0 Gy or 8 Gy irradiation. Scale bar: 20 µm. (D) Western blot analysis for the protein levels of METTL3 in the indicated cells following treatment with 0 Gy or 8 Gy irradiation. (E) RT‐qPCR analysis of LNCAROD expression in the indicated cells with 0 Gy or 8 Gy irradiation. (F) MeRIP‐qPCR analysis of LNCAROD m6A modification in the indicated cells. (G) Apoptosis rates in the indicated cells with 0 Gy or 8 Gy irradiation. (H) Cell cycle distribution in ESCC cells transfected with ASO‐LNCAROD or ASO‐NC with 0 Gy or 8 Gy irradiation. (B–G) cells were collected 24 h after exposure to X‐ray irradiation or no irradiation, while (H) cells were collected 6 h after exposure to X‐ray irradiation or no irradiation. Data are presented as the mean ± SD; (ns *p* > .05, *p < .05, **p < .01, ***p < .001, ****p < .0001).

We subsequently assessed the expression of LNCAROD in KYSE‐150 and KYSE‐150R cells and found that LNCAROD was upregulated in KYSE‐150R cells (Figure 2A). To investigate the impact of short‐term radiation exposure on LNCAROD, we examined LNCAROD expression after treating ESCC cells with a gradient of radiation doses. RT‐qPCR and FISH results confirmed the expression of LNCAROD significantly increased with an increasing radiation dose (Figure 2B and C). These findings imply that LNCAROD could be essential for the acquisition and maintenance of radioresistance. In addition, following 8 Gy radiation, the expression of METTL3 in KYSE‐150 cells was observed to be upregulated, while no significant alteration was detected in the expression of YTHDC1 (Figures 2D and S1H). Silencing METTL3 resulted in decreased expression of LNCAROD and hindered the radiation‐induced increase in LNCAROD levels (Figure 2E). Additionally, KYSE‐150R cells exhibited a significant elevation in the m6A level of LNCAROD in comparison to KYSE‐150 cells (Figure 2F). Taken together, the radiation‐induced upregulation of LNCAROD was due to METTL3 increasing the m6A modification level of LNCAROD.

**FIGURE 3:**
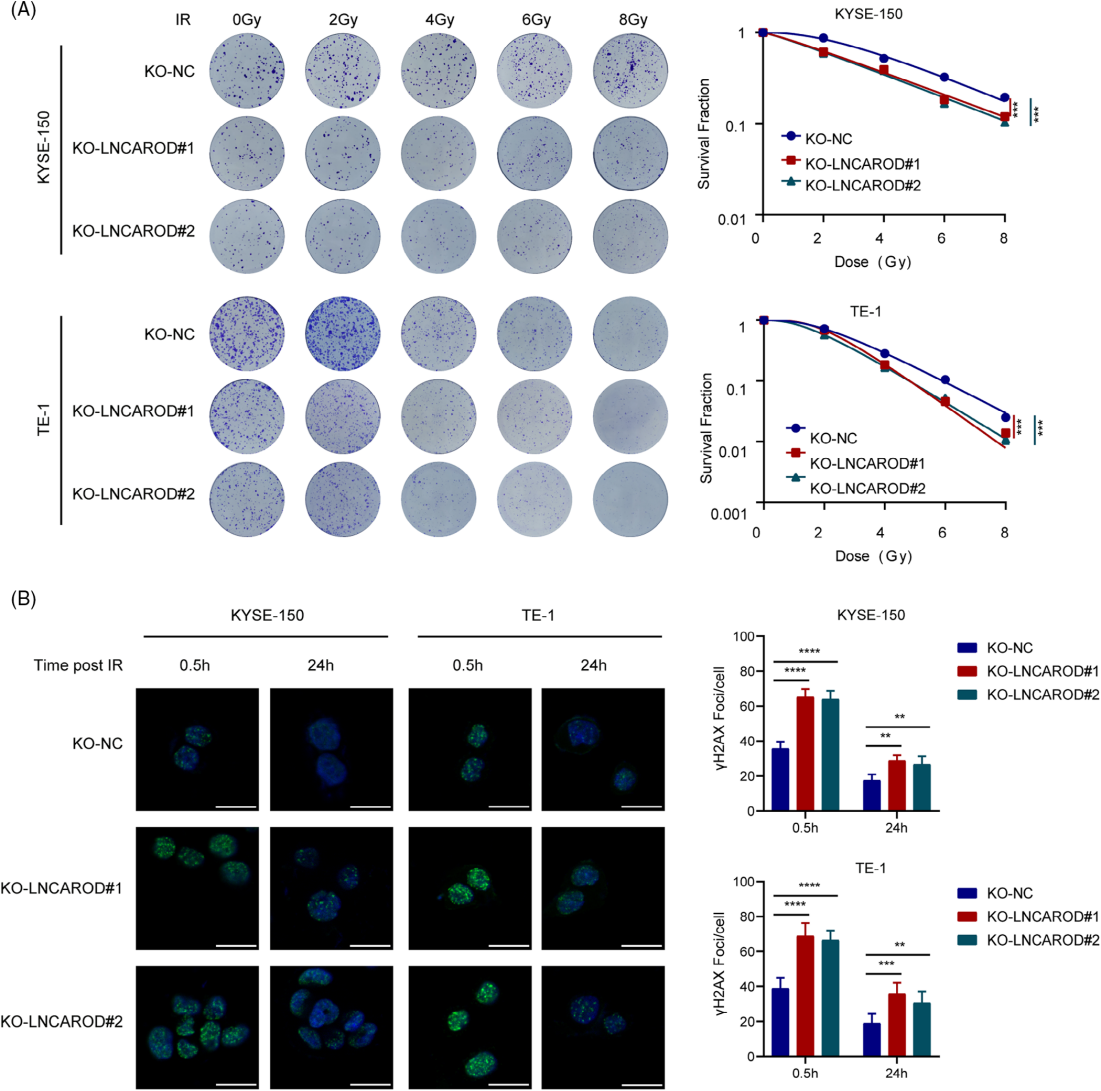
Knockout of LNCAROD enhances the radiosensitivity of ESCC cells. (A) Colony formation assay for survival fraction in indicator cells following exposure to 0, 2, 4, 6 and 8 Gy irradiation. (B) Representative immunofluorescence images of nuclear γH2AX foci (cell nuclei: blue; γH2AX foci: green) in the indicated cells at 0.5 h and 24 h after exposure to 8 Gy irradiation. Scale bar, 20 µm. Data are presented as the mean ± SD; *n* = 3 independent experiments (**p < .01, ***p < .001, ****p < .0001).

We subsequently suppressed LNCAROD expression in KYSE‐150 and TE‐1 cells, with relatively high levels of LNCAROD, by antisense oligonucleotides (ASOs) targeting LNCAROD, and found a notable increase in apoptosis levels upon irradiation exposure in LNCAROD‐deficient cells (Figures 2G and S2A). Regarding the cell cycle distribution, silencing LCNAROD in combination with irradiation resulted in a greater percentage of ESCC cells being arrested in the G_2_/M phase (Figure 2H). We obtained ESCC cells with stable knockout of LNCAROD using CRISPR‐Cas9 technique and performed colony formation assays (Figure S2B and C). Deletion of LNCAROD in ESCC cells resulted in the inhibitions of cell proliferation and colony formation after irradiation (Figure 3A). The γH2AX assay is frequently employed for the identification of DNA damage and double‐strand breaks (DSBs), enabling the assessment of levels of DSB damage and repair.^25^, ^26^ The detection results of γH2AX foci formation revealed a notable rise in the percentage of γH2AX‐positive cells in ESCC cells with LNCAROD gene knockout at  .5 h and 24 h following DNA damage caused by irradiation (Figure 3B). Collectively, these findings indicated that LNCAROD attenuated the DNA damage induced by irradiation and augmented the radioresistance of ESCC cells.

### 3.3 LNCAROD facilitates the protein‐protein interaction between PARP1 and NPM1

**FIGURE 4:**
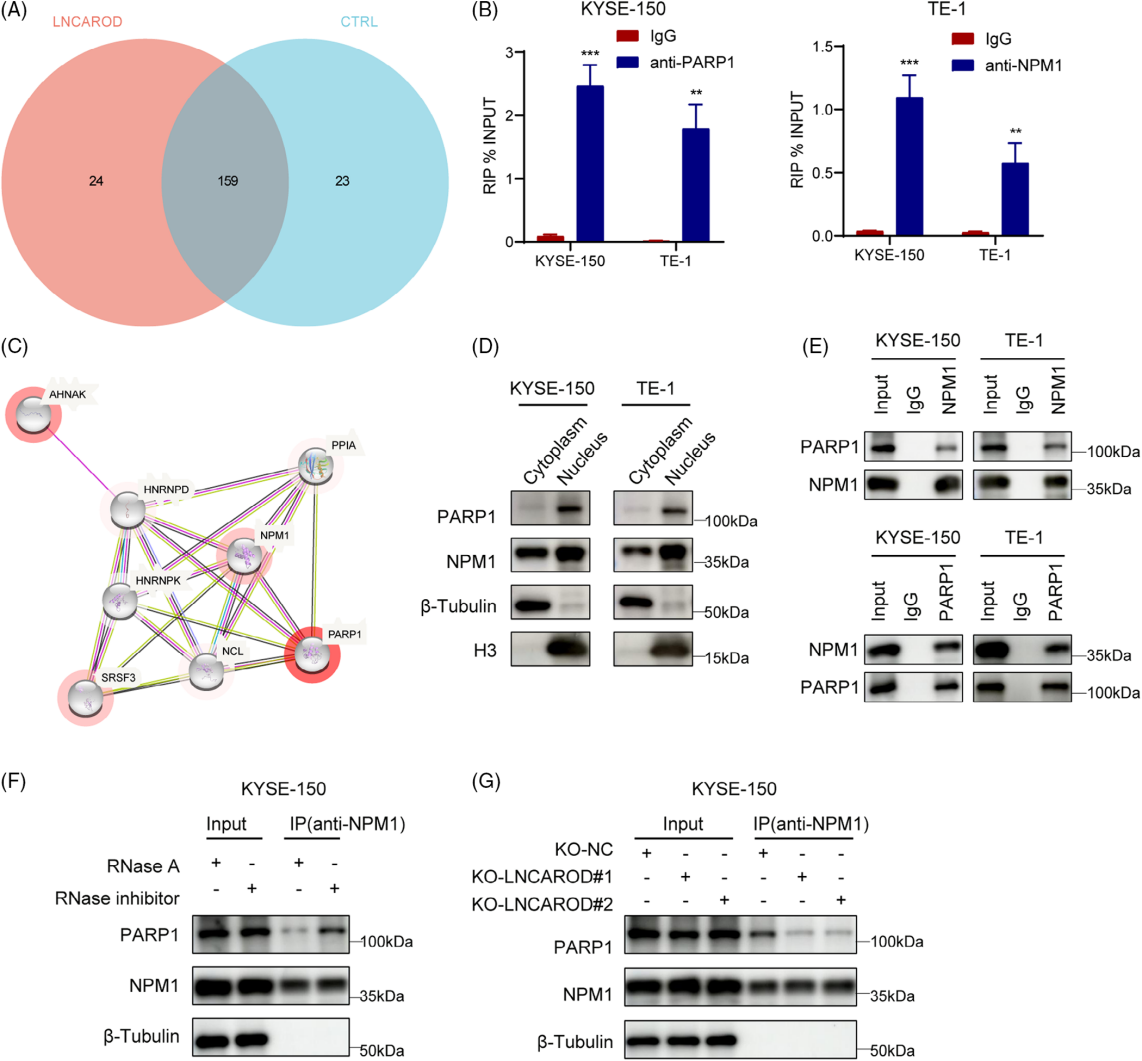
LNCAROD facilitates the protein‐protein interaction between PARP1 and NPM1. (A) The Venn diagram illustrates the quantity of proteins associated with LNCAROD, as identified by ChIRP‐MS analysis. (B) RIP‐qPCR assays shows that PARP1 and NPM1 bind with LNCAROD. (C) STRINGdb protein‐protein network enrichment analysis. (D) Subcellular fractionation and western blot assays show the subcellular location of PARP1 and NPM1 in the ESCC cell lines. (E) Western blot analysis of the interaction between PARP1 and NPM1 after co‐IP assays in KYSE150 and TE‐1 cells. (F, G) Western blot analysis of the interaction between PARP1 and NPM1 following co‐IP assays in the indicated cells. Data are presented as the mean ± SD (**p < .01, ***p < .001).

To elucidate the downstream mechanisms responsible for the radioresistant functions of LNCAROD, we performed a ChIRP‐MS assay to identify LNCAROD binding proteins in KYSE‐150 cells. A total of 24 candidate proteins interacting with LNCAROD were identified in KYSE‐150 cells (Figure 4A and Table S7). Among the top five candidate proteins, poly ADP‐ribose polymerase‐1 (PARP1) and nucleophosmin 1 (NPM1) have been widely reported to be involved in regulating DNA damage repair pathways (Figure S3A and B).^27^, ^28^ Next, RIP assays were conducted to confirm the binding of LNCAROD with PARP1 and NPM1 in ESCC cells (Figure 4B). STRING analysis revealed protein‐protein interactions between PARP1 and NPM1 (Figures 4C and S3C). Subcellular fractionation of ESCC cells showed that PARP1 and NPM1 proteins colocalised in the nucleus (Figure 4D). The endogenous interaction between PARP1 and NPM1 in KYSE‐150 cells and TE‐1 cells was further validated by Co‐IP assays (Figure 4E). However, immunoprecipitation results from cell lysates pretreated with RNase A showed a significantly weaker interaction between PARP1 and NPM1 compared to those pretreated with recombinant RNase inhibitor, indicating the involvement of RNA in this process (Figure 4F). Additionally, knocking out LNCAROD in KYSE‐150 cells attenuated protein‐protein interactions between PARP1 and NPM1 (Figure 4G). These results collectively indicated that LNCAROD acted as a scaffold to enhance the protein‐protein interaction between PARP1 and NPM1.

### 3.4 LNCAROD suppresses ubiquitination degradation of PARP1 in an NPM1‐dependent manner

**FIGURE 5:**
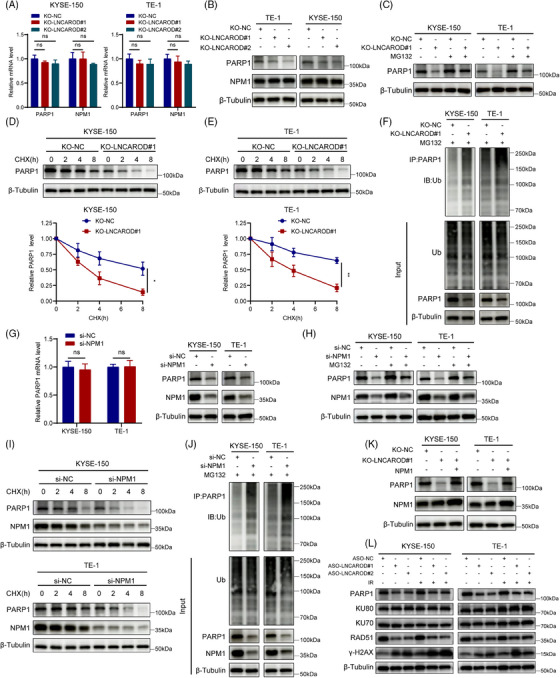
LNCAROD increases PARP1 protein stability in a NPM1‐dependent manner. (A) RT‐qPCR analysis of the mRNA levels of PARP1 and NPM1 in LNCAROD‐depleted cells. (B) Western blot analysis of the protein levels of PARP1 and NPM1 in LNCAROD‐depleted cells. (C) The protein levels of PARP1 in the indicated cells after treatment with 10 µM MG132. (D, E) Western blot analysis of the effect of LNCAROD on PARP1 protein degradation in the presence of 100 µg/mL CHX. (F) Ubiquitination assay of PARP1 in the indicated cells after treatment with MG132 at a concentration of 10 µM. (G) PARP1 mRNA and protein levels in KYSE‐150 and TE‐1 cells transfected with NPM1 specific siRNAs. (H) The protein levels of PARP1 in KYSE‐150 and TE‐1 cells transfected with NPM1 specific siRNAs after treatment with 10 µM MG132. (I) Western blot analysis of the effect of NPM1 on PARP1 protein degradation in the presence of 100 µg/mL CHX. (J) Ubiquitination assay of PARP1 in the indicated cells after treatment with MG132 at a concentration of 10 µM. (K) The effects of NPM1 overexpression on PARP1 protein in the indicated cells. (L) The protein levels of key genes involved in HR and NHEJ pathways in the indicated cells with or without exposure to 8 Gy IR. Data are presented as the mean ± SD; *n* = 3 independent experiments (ns *p* > .05, *p < .05, **p < .01).

Since LNCAROD interacted directly with PARP1 and NPM1, we next examined whether LNCAROD regulates PARP1 or NPM1 expression. Knockout of LNCAROD led to a reduction in the protein abundance of PARP1, while its mRNA expression remained unaffected (Figure 5A and B). Unlike PARP1, both the transcript and protein levels of NPM1 remained unchanged upon depletion of LNCAROD. Therefore, we postulated that LNCAROD may exert regulatory effects on PARP1 at the post‐transcriptional level. Subsequently, we employed the proteasome inhibitor MG132 to determine whether LNCAROD affects PARP1 degradation. The results showed that LNCAROD silencing‐mediated degradation of PARP1 in ESCC cells could be partially reversed by MG132 (Figure 5C). Additionally, we observed that the stability of the PARP1 protein decreased as a result of LNCAROD deficiency (Figure 5D and E). We next evaluated the enrichment of PARP1 ubiquitination through immunoprecipitation, and observed a substantial increase in the ubiquitination level of PARP1 protein following LNCAROD knockout (Figure 5F). These results revealed that LNCAROD enhanced the protein stability of PARP1 by preventing its degradation via the ubiquitin‐proteasome pathway.

We subsequently investigated the potential contribution of NPM1 to the stabilisation of PARP1 protein mediated by LNCAROD in ESCC cells. Silencing NPM1 led to a decrease in PARP1 protein levels in ESCC cells, while its mRNA levels remained unaffected (Figures 5G and S3D). Treatment with MG132 prevented the decrease in PARP1 protein levels in ESCC cells upon NPM1 depletion (Figure 5H). Meanwhile, the half‐life of PARP1 protein was markedly shortened in NPM1‐deficient ESCC cells (Figures 5I and S3E). We further observed a substantial increase in the ubiquitination level of PARP1 protein after silencing NPM1 (Figure 5J). Moreover, the overexpression of NPM1 significantly increased the protein level of PARP1 in LNCAROD knockout KYSE‐150 cells, indicating that NPM1 is crucial for the stabilisation of PARP1 protein mediated by LNCAROD (Figure 5K). Therefore, our findings indicated that LNCAROD promoted the binding between PARP1 and NPM1, thereby preventing the ubiquitin‐proteasome degradation of PARP1 protein. Studies in eukaryotic cells have demonstrated that the HR and non‐homologous end‐joining (NHEJ) pathways are the primary mechanisms for repairing DSBs caused by irradiation.^29^, ^30^ PARP1, as a key sensor for DNA damage repair, can participate in the process of DNA damage repair by recruiting repair effector molecules of HR or NHEJ.^31^ To ascertain the DSB repair pathway regulated by the LNCAROD‐PARP1 axis in ESCC cells, we evaluated the expression levels of pivotal genes implicated in HR and NHEJ pathways. The results revealed that deletion of LNCAROD in ESCC cells significantly reduced PARP1 expression and HR pathway‐related protein RAD51 expression, while levels of NHEJ pathway key proteins such as Ku70 and Ku80 remained unchanged (Figure 5L). Collectively, these results suggested that silencing LNCAROD impaired homologous recombination repair of radiation damage in ESCC cells by reducing PARP1 protein stability.

### 3.5 PARP1 overexpression counteracts the radiosensitising impact caused by LNCAROD knockout

**FIGURE 6:**
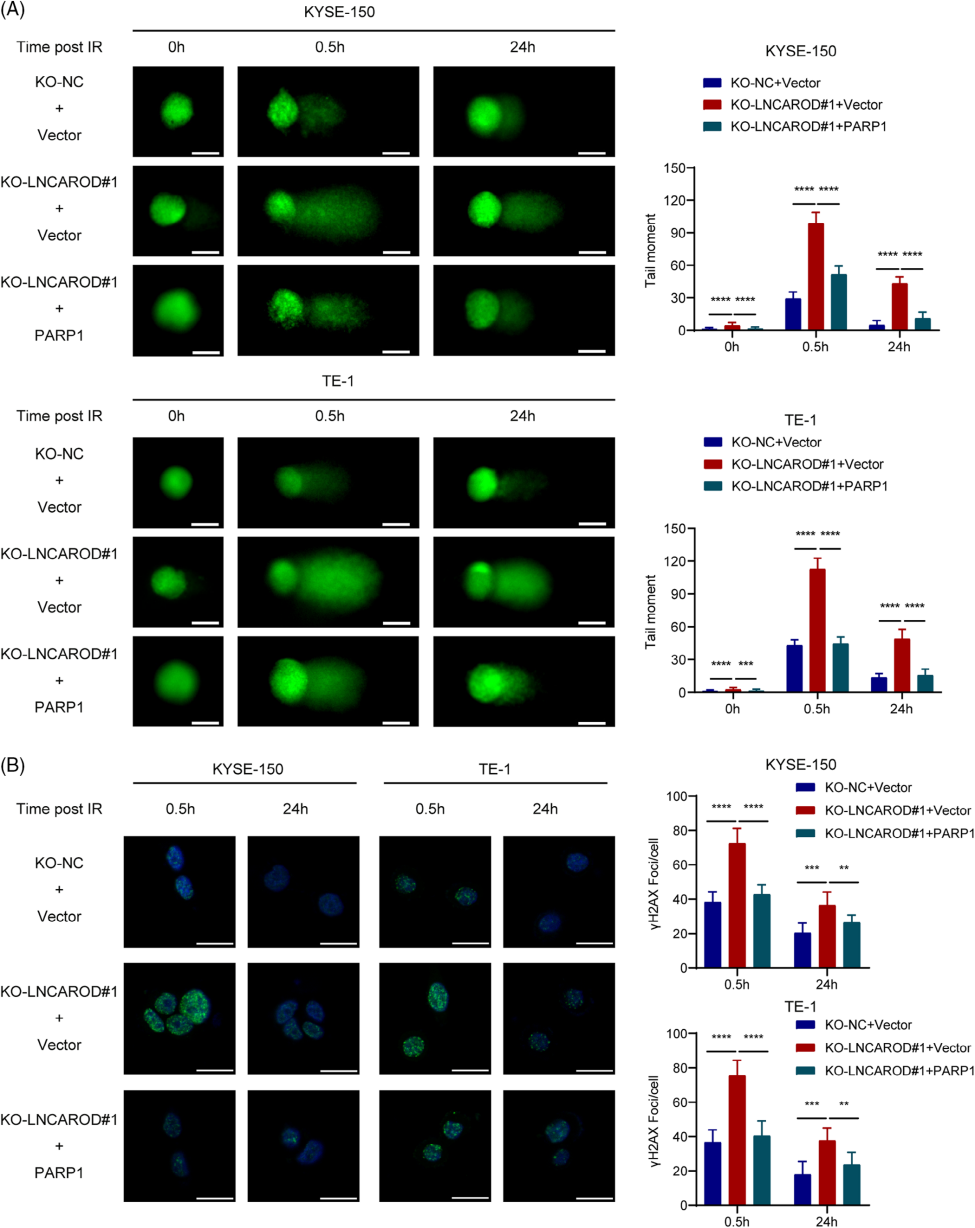
PARP1 overexpression counteracts the radiosensitising impact caused by LNCAROD knockout. (A) Representative images of comet assay and quantitative analysis of tail moment for 8 Gy irradiation‐induced DNA damage in the indicated cells. Scale bar, 20 µm. (B) Representative immunofluorescence images of nuclear γH2AX foci in the indicated cells at 0.5 h and 24 h after exposure to 8 Gy irradiation. Scale bar, 20 µm. Data are shown as the mean ± SD (**p < .01, ***p < .001, ****p < .0001).

We next evaluated whether the radioresistant effect of LNCAROD in ESCC cells was mediated by PARP1. First, reintroduction of a plasmid overexpressing PARP1 led to an elevation in the protein level of PARP1 within ESCC cells with silenced LNCAROD (Figure S3F). Second, we conducted comet assays and found that the tail moments of LNCAROD knockout cells prolonged significantly within 24 h after radiation compared to the control group cells, indicating an increase in DNA damage. Moreover, overexpression of PARP1 effectively reversed the effects of LNCAROD deficiency on DNA damage induced by irradiation exposure, suggesting that LNCAROD regulated PARP1 to alleviate irradiation‐induced DNA damage (Figure 6A). Immunofluorescence staining results revealed a significant augmentation in radiation‐induced γH2AX focal formation upon depletion of LNCAROD, which was effectively counteracted by the introduction of exogenous expression of PARP1 (Figure 6B).

**FIGURE 7:**
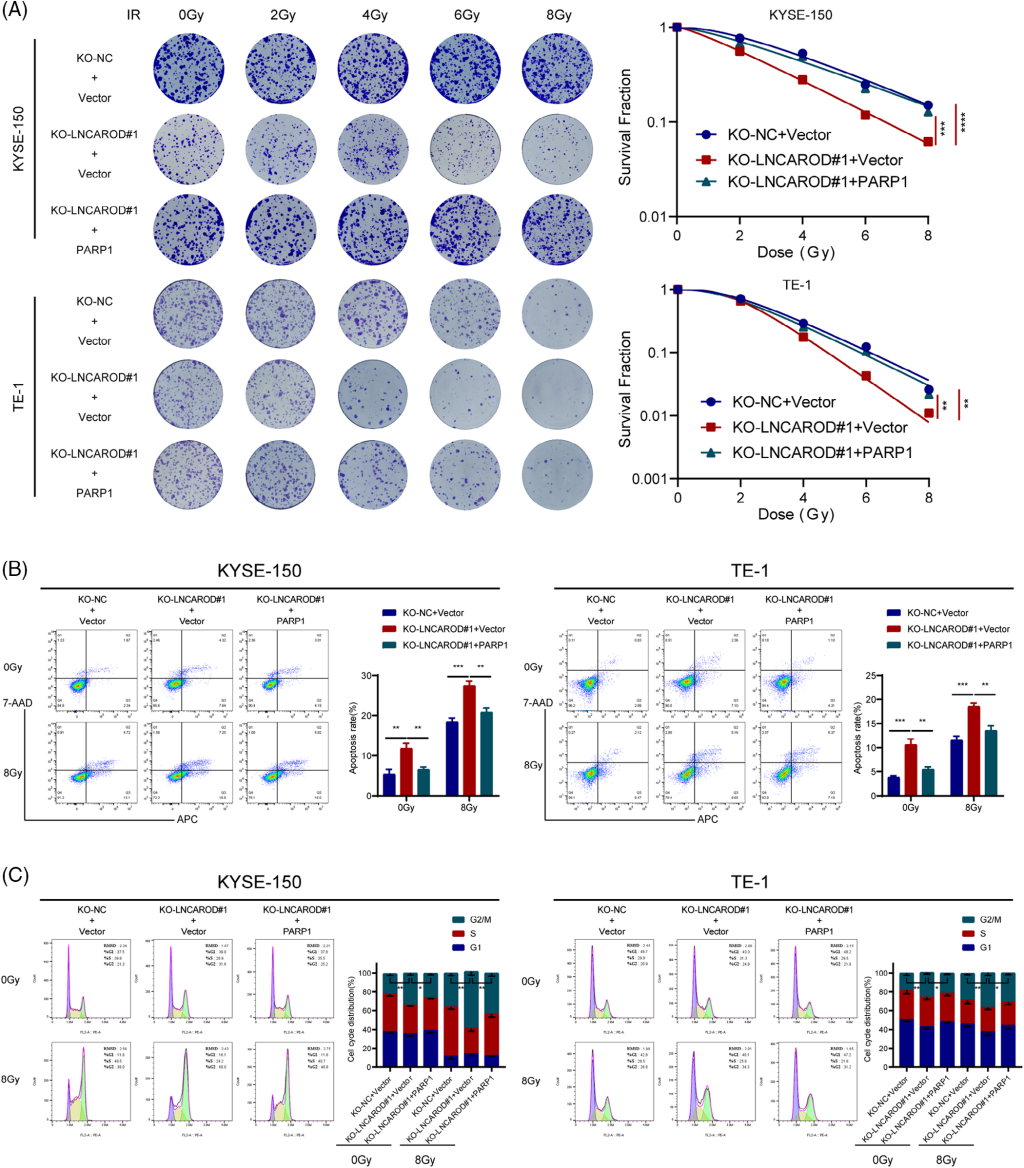
PARP1 overexpression restores the radiosensitising effect of LNCAROD deficiency in vitro. (A) Colony formation assay for survival fraction in indicator cells following exposure to 0, 2, 4, 6 and 8 Gy irradiation. (B) Apoptosis rates in the indicated cells with 0 Gy or 8 Gy irradiation. (C) Cell cycle distribution in the indicated cells with 0 Gy or 8 Gy irradiation. Data are shown as the mean ± SD (*p < .05, **p < .01, ***p < .001, ****p < .0001).

The results from the colony formation experiments demonstrated that upregulating PARP1 expression effectively rescued the survival fraction of LNCAROD‐deficient cells after irradiation treatment, thereby counteracting the radiosensitising impact induced by depletion of LNCAROD (Figure 7A). Additionally, flow cytometry analysis showed that silenced LNCAROD significantly enhanced the levels of cell apoptosis induced by radiation, while upregulation of PARP1 mitigated the pro‐apoptotic impact resulting from LNCAROD knockout (Figure 7B). Cell cycle assay revealed that overexpression of PARP1 significantly attenuated the increase in G_2_/M phase ratio in LNCAROD knockout cells after irradiation treatment (Figure 7C). Overall, these findings suggested that LNCAROD silencing impaired the radioresistance of ESCC cells, whereas overexpression of PARP1 reversed this impairment.

### 3.6 Knockout of LNCAROD facilitates radiosensitivity of ESCC cells in vivo

**FIGURE 8:**
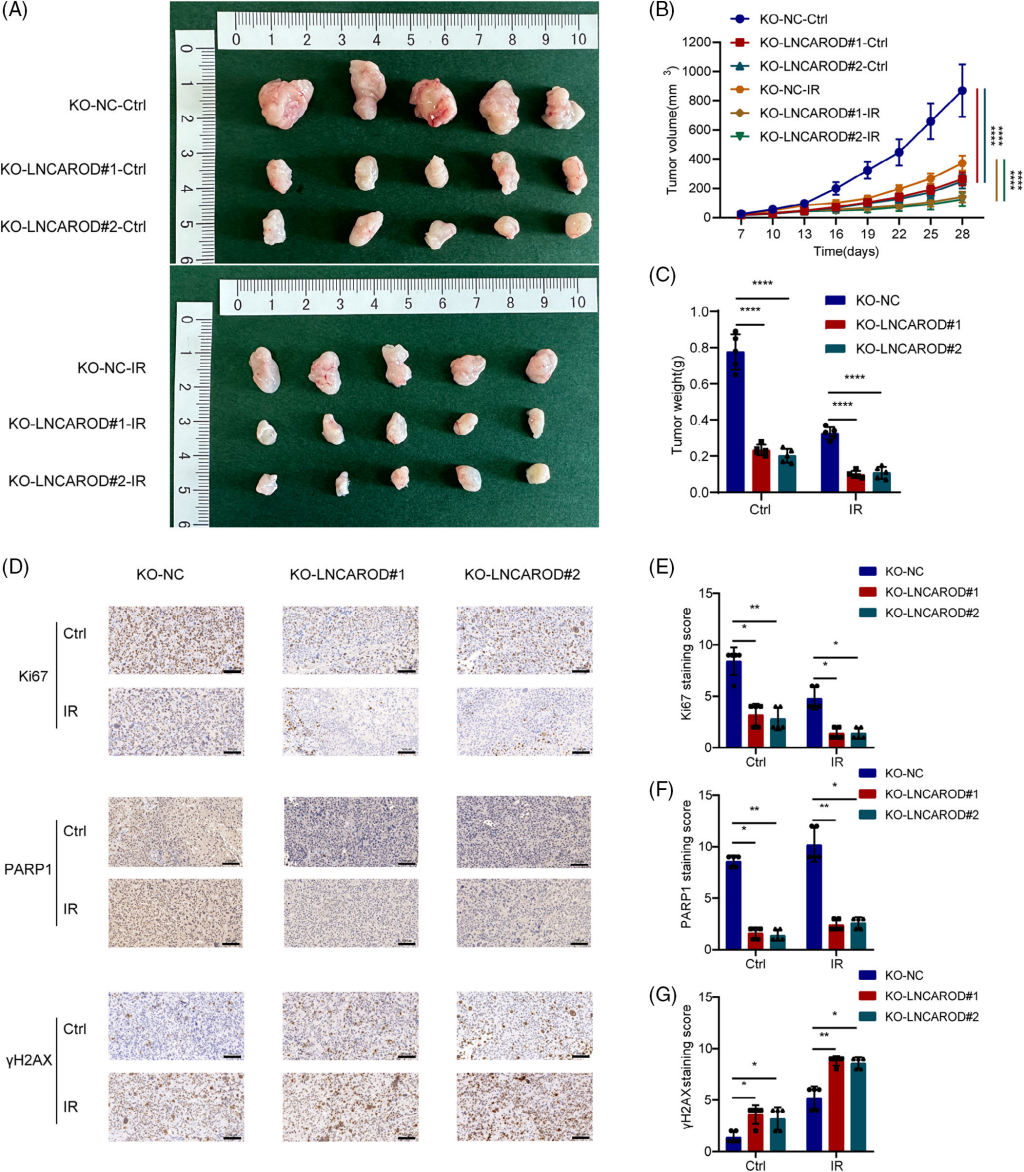
LNCAROD depletion enhances the radiosensitivity of ESCC cells in vivo. (A–C) Photographs of excised subcutaneous tumours, tumours growth curves, and tumours weight of KYSE‐150 cells stably transfected with KO‐NC, KO‐LNCAROD#1 or KO‐LNACROA#2 and exposed to irradiation or not. (D–G) IHC pictures and staining scores for Ki67, PARP1 and γH2AX protein expression in excised subcutaneous tumours from different groups. Scale bar, 100 µm. Data are shown as the means ± SD (*p < .05, **p < .01, ***p < .001, ****p < .0001).

To further confirm the radiosensitising effect of LNCAROD depletion in ESCC cells in vivo, we constructed xenograft models by subcutaneous inoculation of ESCC cells into BALB/c nude mice, which were then irradiated and analysed. As indicated in Figure 8A‐C, compared with the negative control group, the tumour growth was slower and the tumour size and weight were decreased in the LNCAROD knockout group, especially after irradiation treatment, suggesting that tumours in the LNCAROD deletion group exhibited increased sensitivity to irradiation. Furthermore, IHC analyses revealed that Ki67 and PARP1 protein levels were significantly reduced and γH2AX protein expression was strikingly increased in ESCC xenograft tumours with LNCAROD knockout group, especially when combined with irradiation treatment (Figure 8D‐G). These results illustrated that the silencing LNCAROD reduced the expression level of PARP1, thereby enhancing the radiosensitivity of ESCC cells in vivo by impairing DNA damage repair.

## 4 DISCUSSION

**FIGURE 9:**
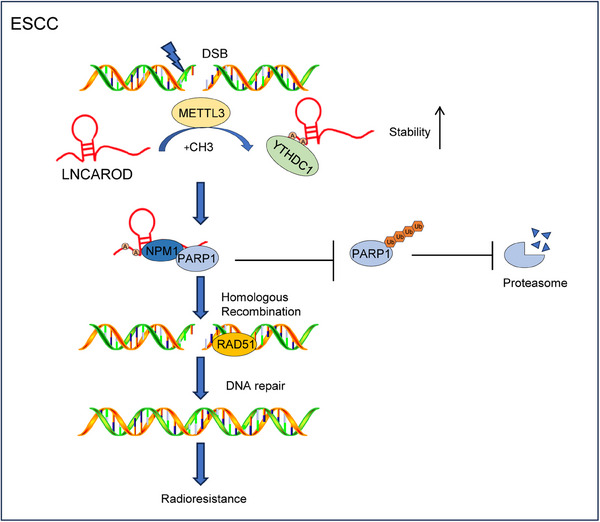
A schematic model illustrating the mechanism by which LNCAROD enhances the radioresistance of ESCC. METTL3‐mediated m6A modification enhances the stability of LNCAROD in a YTHDC1‐dependent manner. LNCAROD prevents ubiquitination degradation of PARP1 protein by facilitating PARP1‐NPM1 interaction, thereby contributing to homologous recombination‐mediated DNA double‐strand breaks repair and enhancing the radiation resistance of ESCC cells.

Radiotherapy is a standard treatment for patients with locally advanced or inoperable ESCC.^32^ However, radioresistance remains a significant barrier that hinders the enhancement of radiotherapy effectiveness, and identifying possible targets to counteract radioresistance of ESCC represents a promising area of research.^33^ Here, we identified LNCAROD as a novel METTL3‐mediated lncRNA that enhanced radioresistance in ESCC cells and was post‐transcriptionally stabilised by YTHDC1. Moreover, we confirmed that LNCAROD hinders ubiquitin‐proteasome degradation of PARP1 protein by promoting PARP1‐NPM1 interaction, thereby contributing to HR‐mediated DSBR and enhancing the radiation resistance of ESCC cells. Figure 9 shows our working model. These findings highlight the importance of m6A‐modified LNCAROD in ESCC radioresistance.

Increasing evidence indicates that lncRNAs are significantly involved in the development of radioresistance in malignant tumours, such as ESCC.^34^, ^35^ As a newly identified lncRNA, LNCAROD has been reported as an oncogene in gastric cancer and head and neck squamous cell carcinoma.^36^, ^37^ Here, we demonstrated the upregulation of LNCAROD in ESCC cell lines and radioresistant cell lines when compared to their corresponding controls. Besides, the expression of LNCAROD was observed to be upregulated following exposure to ionising radiation. m6A is the most prevalent modification found in eukaryotic mRNAs and lncRNAs, playing a pivotal role in post‐transcriptional gene regulation.^38^ An increasing body of evidence has demonstrated that the dynamic alterations of m6A modification offer a novel avenue for enhancing tumour radiosensitivity.^39^ Altered METTL3 expression can affect the expression of DNA damage response (DDR)‐related genes and impair the repair of radiation‐induced DNA damage, rendering cancer cells more resistant to irradiation‐induced cytotoxicity.^40^ Our study provides the first evidence that the expression level of METTL3 and its mediated m6A modification are elevated in radioresistant ESCC cells compared to parental cells. Additionally, we observed that METTL3 mediated the m6A modification of LNCAROD and enhanced LNCAROD expression in an YTHDC1‐dependent manner. Furthermore, functional experiments demonstrated that the presence of LNCAROD increased the survival rate of ESCC cells subjected to varying levels of ionising radiation, suppressed irradiation‐induced apoptosis, reduced the percentage of cells in G_2_/M phase, and attenuated γH2AX foci formation. We acknowledge that the survival fractions in our study at 6 Gy were higher than those reported in some other studies, which could be attributed to the specific characteristics of the cell lines used. This resistance presents a significant challenge in improving the efficacy of radiotherapy for ESCC. Despite this resistance, our approach targeting LNCAROD showed a measurable, albeit moderate, sensitisation effect. Based on these findings, we have demonstrated for the first time that m6A‐modified LNCAROD reduces radiosensitivity in ESCC, highlighting it as a potential therapeutic target to alleviate radioresistance.

After screening by ChIRP‐MS assay, we discovered PARP1 and NPM1 as downstream proteins that specifically bind to LNCAROD. PARP1, the most abundant member of the PARP enzyme family, is recognised as an early sensor of DNA damage, facilitating efficient DNA repair processes and maintaining genomic stability.^41^ NPM1, belonging to the nucleophosmin/nucleoplasmin family, plays indispensable roles in maintaining genome stability and responding to various forms of DNA damage.^42^, ^43^ Here, we identified an important mechanism by which LNCAROD prevents ubiquitin‐proteasome degradation of PARP1 protein by facilitating PARP1‐NPM1 interaction. First, we confirmed the interaction between PARP1 and NPM1 protein in ESCC cells, which is consistent with previous findings in breast cancer cells.^44^ Second, we found that silencing LNCAROD weaken the interaction of PARP1 and NPM1, leading to increased ubiquitination degradation of PARP1 protein. Ionising radiation exposure causes a wide variety of lethal DNA damage, especially DSBs. The HR and NHEJ pathways are the primary mechanisms employed by eukaryotic cells to repair DSBs caused by ionising radiation. Several studies have indicated that PARP1 is essential for addressing severe DNA damage and facilitating intricate repair of double‐strand breaks, mainly through the HR or NHEJ pathways. The results of this study confirm that silencing LNCAROD impairs homologous recombination repair of radiation damage in ESCC cells by reducing the stability of the PARP1 protein. We also observed that the deletion of LNCAROD in ESCC cells augmented irradiation‐induced DNA damage, G_2_/M phase arrest, and cell apoptosis, all of which were significantly mitigated by PARP1 overexpression. Moreover, subcutaneous xenograft model confirmed that LNCAROD‐deficient tumours exhibited heightened sensitivity to ionising radiation. These findings indicate that LNCAEOD targets PARP1 to modulate the repair of DNA double‐strand breaks and enhance radioresistance in ESCC cells.

## 5 CONCLUSIONS

Overall, our study indicates that m6A‐modified LNCAROD confers radioresistance of esophageal squamous cell carcinoma through stabilising PARP1, thus providing a new perspective and clue to alleviate radioresistance.

## AUTHOR CONTRIBUTIONS

Hongbing Ma, Xixi Zhao and Xiaozhi Zhang conceived and designed the research. Xiaobo Shi performed experiments. Xinran Huang, Ruijuan Zhang, Shupei Pan, Shan Huang and Yuchen Wang assisted with the statistical analyses. Xiaobo Shi and You Li wrote the first draft of the article. Yue Ke, Wei Guo, Xiaoxiao Liu, Yu Hao, Xixi Zhao and Hongbing Ma reviewed and edited the manuscript. Xiaozhi Zhang, Xu Zhao, Yuchen Sun and Jing Li provided reagents and materials/gave intellectual input. All authors have read and agreed to the final version of the manuscript.

## CONFLICT OF INTEREST STATEMENT

The authors declare that they have no conflicts of interest.

### ETHICS STATEMENT

The research on the animal was authorised by the Laboratory Animal Center of Xi'an Jiaotong University and all applicable norms and laws were followed.

## Supporting information



Supporting Information

Supporting Information

Supporting Information

Supporting Information

Supporting Information

Supporting Information

Supporting Information

Supporting Information

Supporting Information

Supporting Information

Supporting Information

## Data Availability

All data generated was shown in this manuscript. TCGA‐ESCC (https://www.cancer.gov/tcga) and the data sets of GSE53625 from GEO (https://www.ncbi.nlm.nih.gov/geo/) are publicly open and available.
