# Supplementary material for: N6‐methyladenosine‐mediated upregulation of LNCAROD confers radioresistance in esophageal squamous cell carcinoma through stabilizing PARP1
Source: Clin Transl Med. 2024 Oct 5;14(10):e70039. doi: 10.1002/ctm2.70039 (PMC11452732; doi:10.1002/ctm2.70039)
Supplement: Supplementary file 1 — Supporting Information [file CTM2-14-e70039-s003.docx]

Supplementary Figures

**
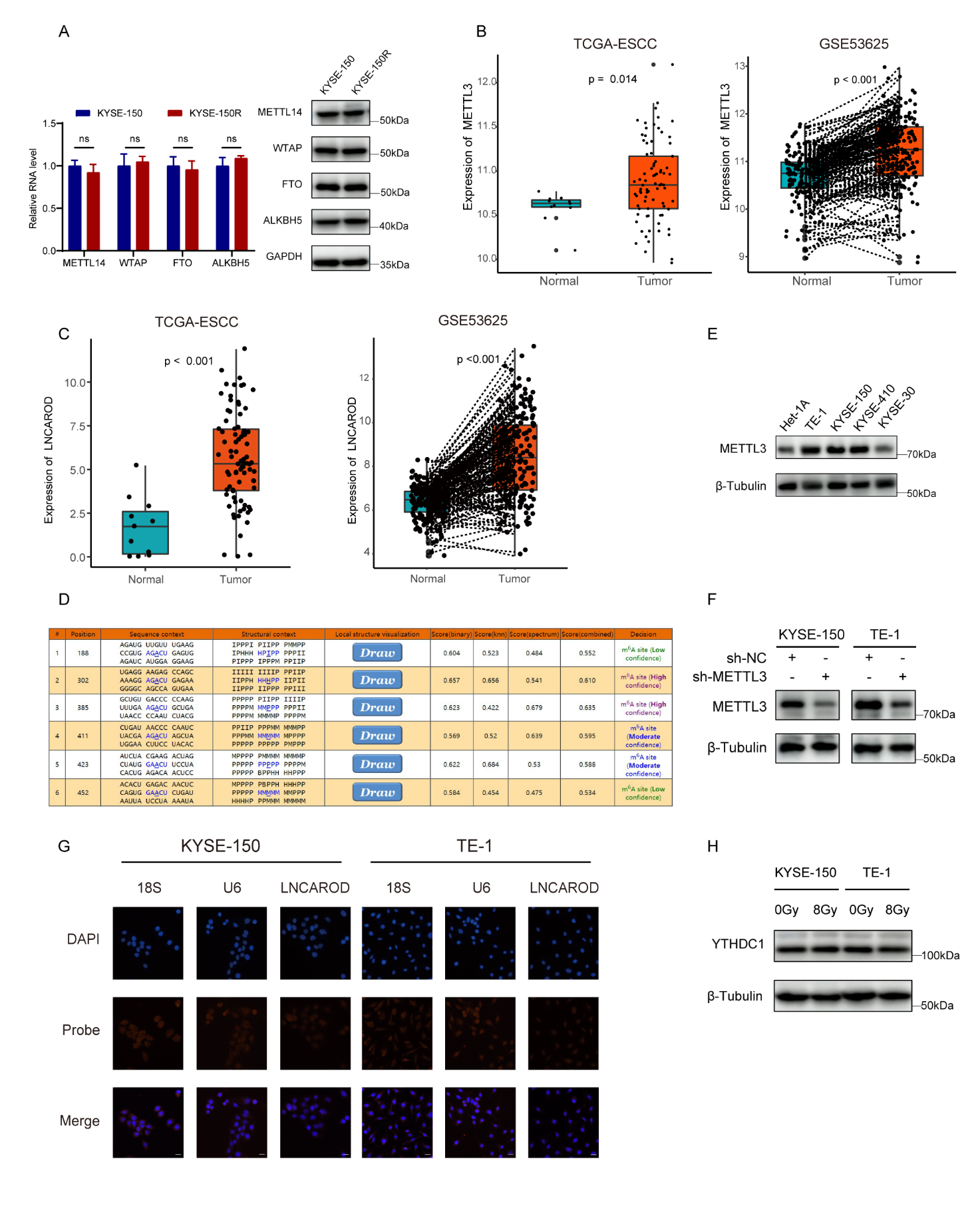
**

**Supplementary Figure 1.** METTL3 stabilizes LNCAROD by m6A demethylation. (A) RT-qPCR and Western blot analysis of mRNA and protein expression for METTL14, WTAP, FTO and ALKBH5 in radioresistant and parental ESCC cell lines. (B-C) The expression patterns of METTL3 and LNCAROD in TCGA-ESCC cohort (ESCC tissue (n = 81) vs normal tissues (n = 11)) and GSE53625 (ESCC tissue vs paired normal tissues (n = 179)). (D) The m6A modification sites prediction results of LNCAROD from the online SRAMP database. (E) The protein expression levels of METTL3 were examined in ESCC cells using Western blot. (F) The protein expression levels of METTL3 in KYSE-150 and TE-1 cells stably transfected with sh-NC or sh-METTL3 plasmids are detected by western blot assays. (G) FISH analysis of LNCAROD in KYSE-150 and TE-1 cells. (The nuclei were stained with DAPI. U6 and 18S rRNA are used as nuclear and cytoplasmic markers, respectively. Scale bar: 20 µm.). (H) Western blot analysis for the protein levels of YTHDC1 in the indicated cells following treatment with 0 Gy or 8 Gy irradiation. Data are presented as the mean ± SD; n = 3 independent experiments. (ns p > 0.05).

**
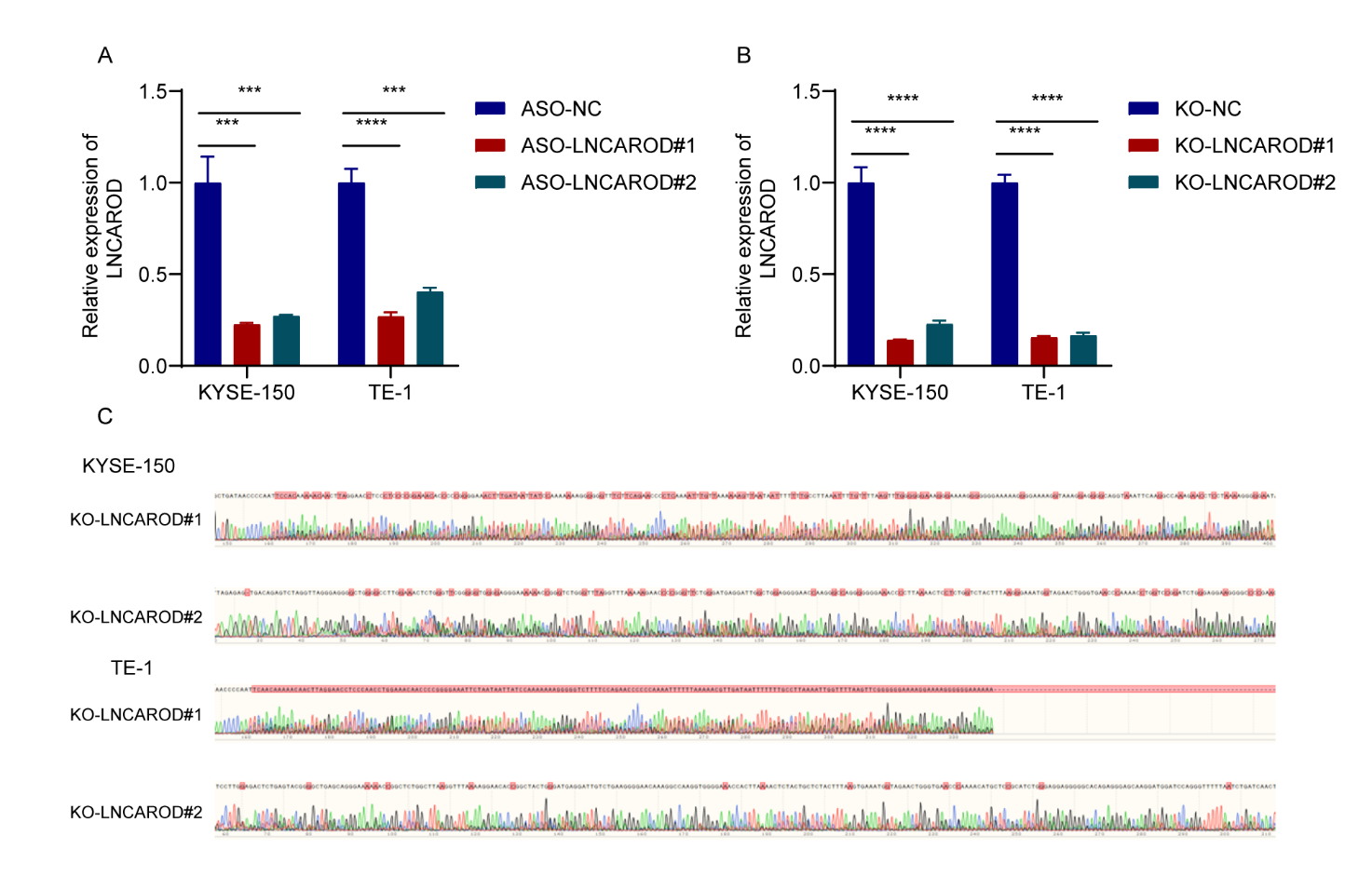
**

**Supplementary Figure 2.** Silencing LNCAROD enhances the radiosensitivity of ESCC cells. (A) The relative expression levels of LNCAROD in KYSE-150 and TE-1 cells transfected with ASO-NC, ASO-LNCAROD#1 or ASO-LNCAROD#2 are determined by RT-qPCR. (B) The relative expression levels of LNCAROD in KYSE-150 and TE-1 cells stably transfected with KO-NC, KO-LNCAROD#1 or KO-LNCAROD#2 are determined by RT-qPCR. (C) CRISPR mediates LNCAROD genome editing in KYSE-150 and TE-1 cells. Data are presented as the mean ± SD; n = 3 independent experiments. (*** p < 0.001, **** p< 0.0001).

**
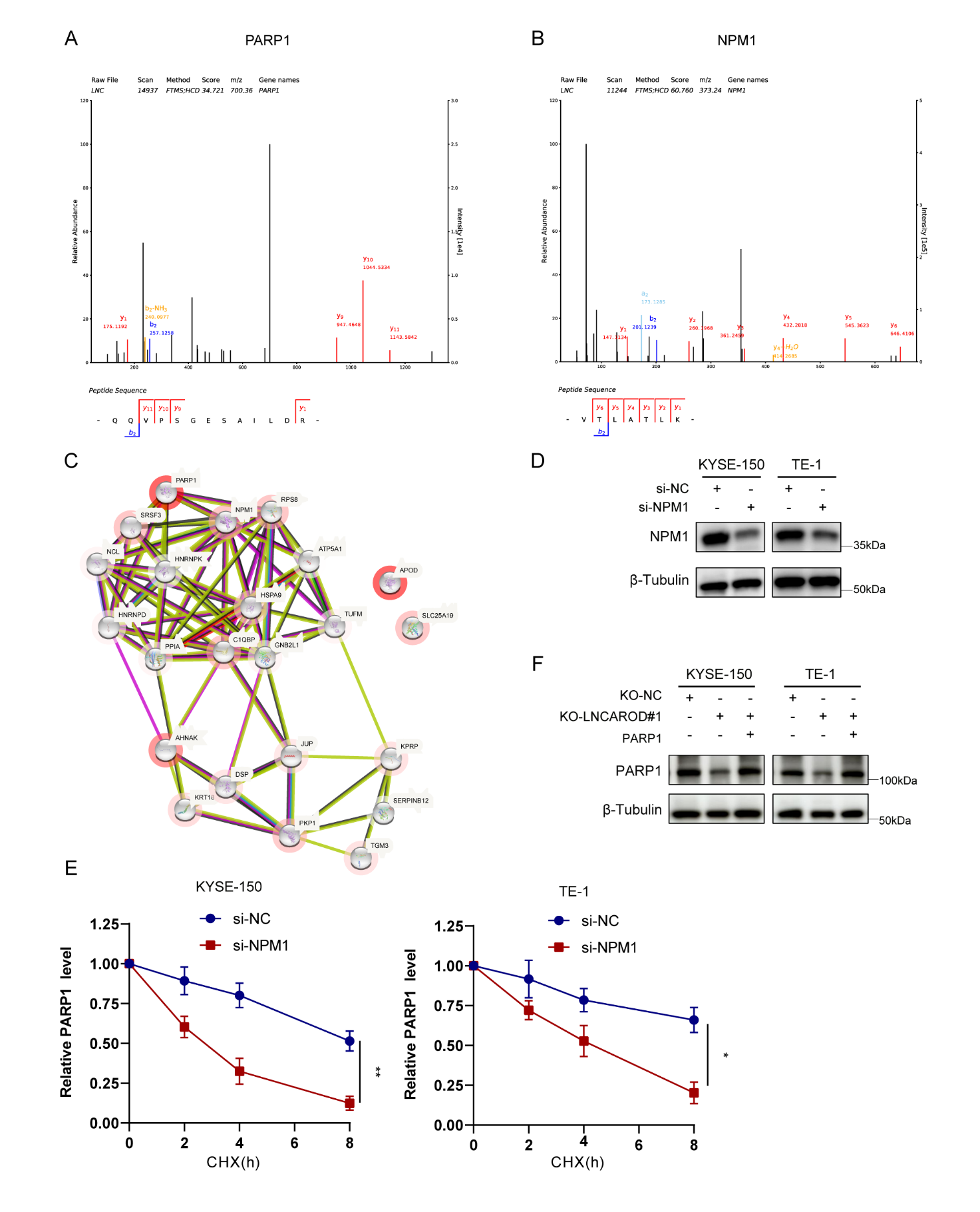
**

**Supplementary Figure 3.** LNCAROD facilitates the protein-protein interaction between PARP1 and NPM1. (A-B) LNCAROD junction-specific peptides of PARP1 (A) or NPM1 (B) are identified in KYSE150 cells by conducting ChIRP-MS. (C) Network enrichment analysis of LNCAROD binding proteins. (D) The protein expression levels of NPM1 in KYSE-150 and TE-1 cells transfected with si-NC or si-NPM1 are detected by western blot assays. (E) The greyscale quantification analysis of the effect of NPM1 on PARP1 protein degradation in the presence of 100 μg/mL CHX. (F) The protein levels of PARP1 are measured in the indicated cells treated with PARP1 plasmids. Data are presented as the mean ± SD; n = 3 independent experiments. (* p < 0.05, ** p < 0.01).
