# Supplementary figures and images for: N6‐methyladenosine‐mediated upregulation of LNCAROD confers radioresistance in esophageal squamous cell carcinoma through stabilizing PARP1
Source: Clin Transl Med. 2024 Oct 5;14(10):e70039. doi: 10.1002/ctm2.70039 (PMC11452732; doi:10.1002/ctm2.70039)

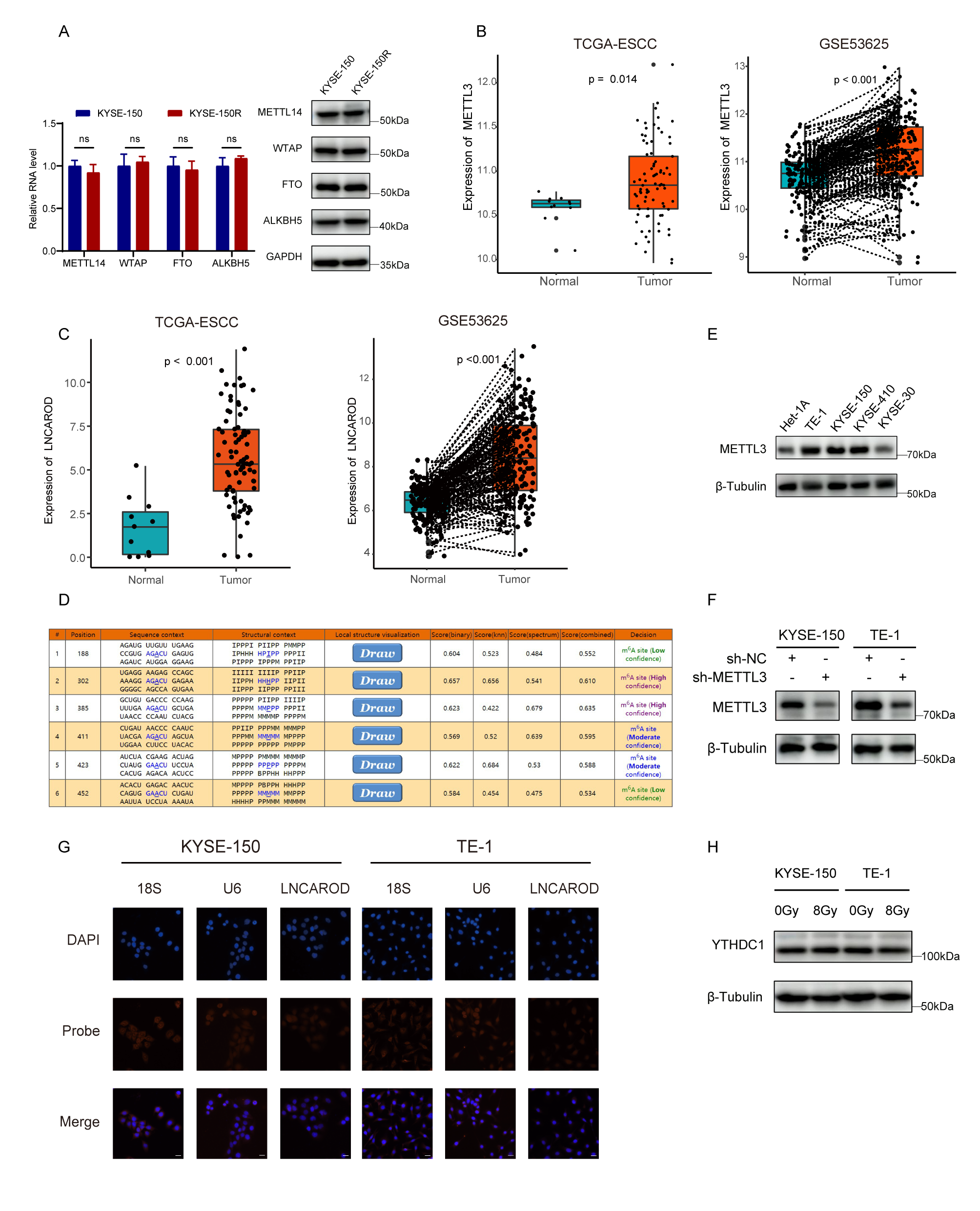

Supplement: Supplementary file 2 — Supporting Information [file CTM2-14-e70039-s008.tif]

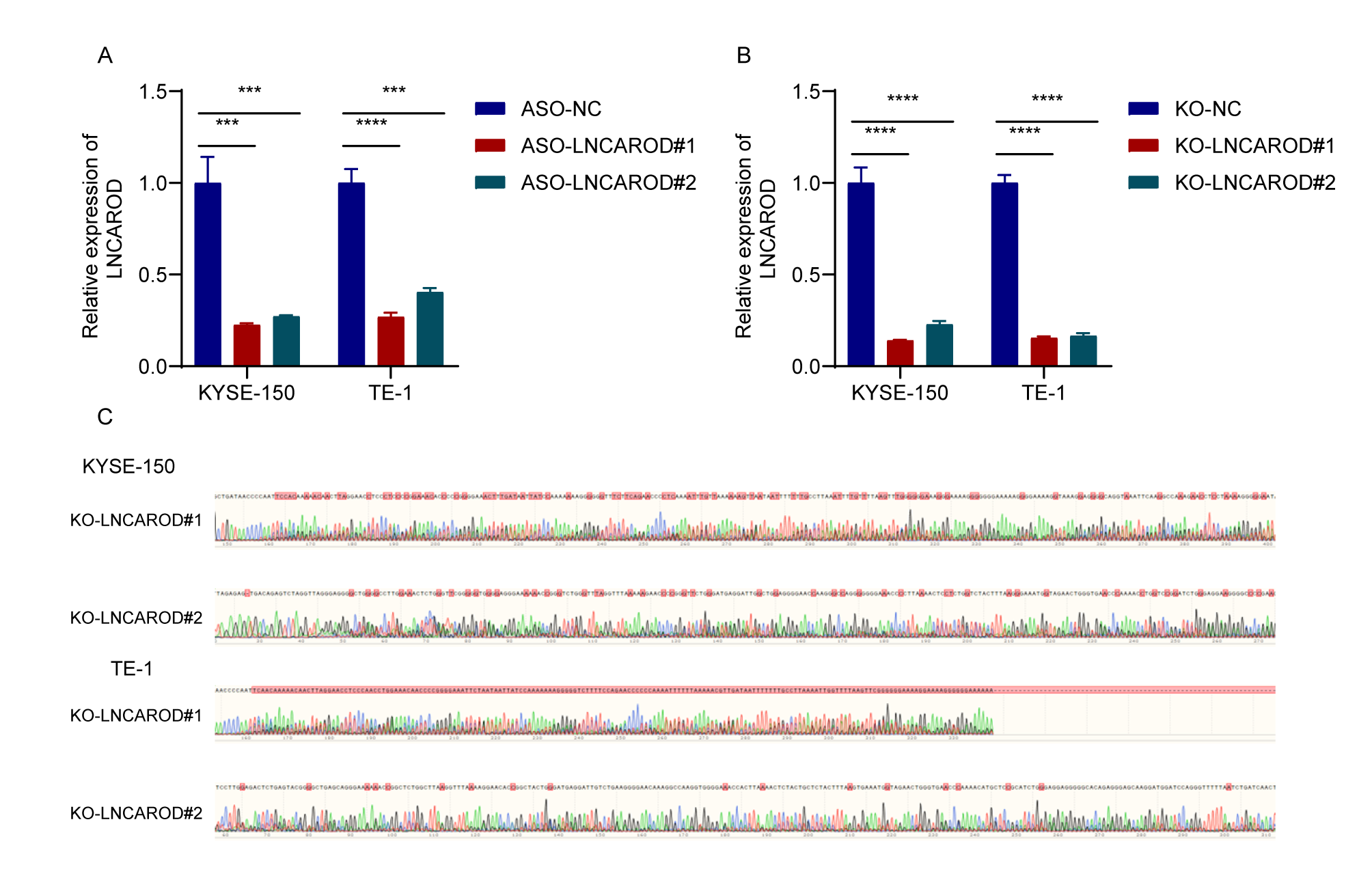

Supplement: Supplementary file 3 — Supporting Information [file CTM2-14-e70039-s010.tif]

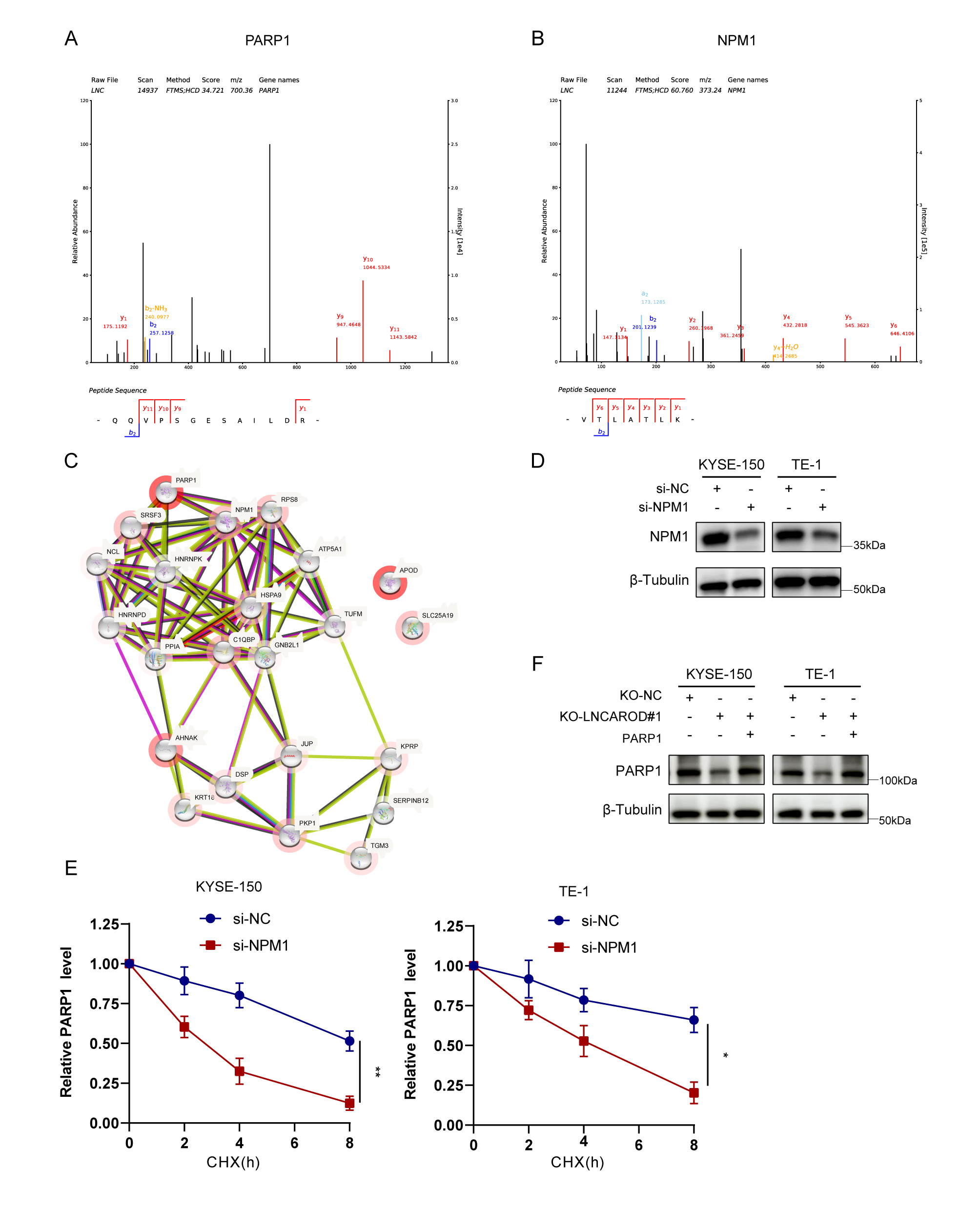

Supplement: Supplementary file 4 — Supporting Information [file CTM2-14-e70039-s002.tif]
